# Enhancing nitrilase production from *Fusarium proliferatum* using response surface methodology

**DOI:** 10.1186/2193-1801-2-290

**Published:** 2013-07-01

**Authors:** Farnaz Yusuf, Asha Chaubey, Arvind Raina, Urmila Jamwal, Rajinder Parshad

**Affiliations:** Fermentation Technology Division, Indian Institute of Integrative Medicine, Canal Road, Jammu Tawi, 180001 India

**Keywords:** Nitrilase, *Fusarium proliferatum*, Design of experiments, Central composite design, Response surface methodology

## Abstract

The individual and interactive effects of three independent variables i.e. carbon source (glucose), nitrogen source (sodium nitrate) and inducer (ϵ-caprolactam) on nitrilase production from *Fusarium proliferatum* were investigated using design of experiments (DOE) methodology. Response surface methodology (RSM) was followed to generate the process model and to obtain the optimal conditions for maximum nitrilase production. Based on central composite design (CCD) a quadratic model was found to fit the experimental data (*p*<0.0001) and maximum activity of 59.0U/g biomass was predicted at glucose concentration (53.22 g/l), sodium nitrate (2.31 g/l) and ϵ-caprolactam (3.58 g/l). Validation experiments were carried out under the optimized conditions for verification of the model. The nitrilase activity of 58.3U/g biomass obtained experimentally correlated to the predicted activity which proves the authenticity of the model. Overall 2.24 fold increase in nitrilase activity was achieved as compared to the activity before optimization (26U/g biomass).

## Introduction

Nitriles, organic compounds containing (−CN) functional group, are widespread in the environment. Naturally occurring nitriles mainly comprise cyanoglycosides**,** cyanolipids and phenylacetonitrile (Marron et al. 2012) that are produced as defensive metabolites in plants. In the chemical industry, nitriles are extensively used as feedstocks, solvents, polymers, pharmaceuticals, pesticides and drug intermediates. Nitriles are also important intermediates in the organic synthesis of amides, amines, carboxylic acids, esters and ketones (Gong et al. 2012; Kobayashi and Shimizu 2000). Bio- and chemo-catalyzed transformations of nitrile substrates lead to the formation of several industrially important amides and organic acids, such as acrylamide, nicotinamide, acrylic and mandelic acids, methacrylic acid (Xue et al. 2011). The biocatalytic route is of particular interest because the nitrile-degrading enzymes like nitrilases, amidases and nitrile hydratases (−NHases) can convert diverse nitrile substrates into various amides and carboxylic acids under relatively moderate process conditions with excellent chemo-, regio- and stereoselectivities (Babu and Shilpi 2010). Nitrilase-mediated biocatalysis has gained substantial attention for the development of sustainable green technologies for the production of selective and complex active ingredients in pharmaceuticals and agrochemicals. Nitrilases catalyse direct cleavage of nitriles to corresponding acids and ammonia (Gong et al. 2012).

Since the first report on nitrilase appeared in 1960s, purification and characterization of nitrilases are being regularly published (Petříčková et al. 2012; Vejvoda et al. 2010 Gupta et al. 2010; Thuku et al. 2009; O’Reilly and Turner 2003). Bacteria have been extensively studied and exploited as source of nitrile hydrolysing enzymes, as compared to eukaryotic organisms (Gong et al. 2012; Sharma et al. 2011; Schreiner et al. 2010; Vejvoda et al. 2010; Martínková et al. 2009; Shen et al. 2009; Zhu et al. 2008; Kaplan et al. 2006a; Banerjee et al. 2002). Filamentous fungi are known to be the rich source of nitrile hydrolysing enzymes especially nitrilases, however, they have not been exploited extensively. Therefore, there is great need to explore them for nitrilase production in detail. The known fungal genera reported for nitrilase production include *Fusarium, Gibberella, Aspergillus, and Penicillium* etc. (Wu et al. 2013; Gong et al. 2012; Martínková et al. 2009). As shown in Table 1, *Aspergillus niger* K10 has been reported to produce nitrilase activity of 100U/L culture (Kaplan et al. 2006a), *Fusarium solani* IMI 196840 produced 1500U/L culture (Vejvoda et al. 2010), *Fusarium solani* O1 has been reported to produce maximum nitrilase activity i.e. 3000U/L culture (2008). Recently, nitrilase from *Fusarium proliferatum* ZJB-09150 has been reported to produce 0.322U/mg DCW for 3-cyanopyridine hydrolysis (Jin et al. 2012). This strain has nitrilase as well as nitrile hydratase activity and therefore has potential applications in organic acids/amide synthesis. However, specificity and selectivity of an enzyme is essential for the development of a successful biotransformation processes to reduce overall cost. Therefore, more selective strains such as *Fusarium solani* and *Fusarium oxysporum* with only nitrilase activity would be a preferred choice for such applications (Martínková et al. 2009).

**Table 1:** **Some fungal strains reported for nitrilase production**

Filamentous fungi	Nitrilase activity (U/l culture)	Substrate	Reference
*Fusarium solani* IMI 196840	1500	Benzonitrilile	Vejvoda et al. 2010
*Fusarium solani* O1	>3000	Benzonitrile	Vejvoda et al. 2008
*Fusarium oxysporium* CCF 1414	119.7	Benzonitrile	Kaplan et al. 2006b
*Fusarium oxysporium* CCF 483	17.6	Benzonitrile	Kaplan et al. 2006b
*Fusarium solani* f. sp. *melonis*	160	Benzonitrile	;Martínková et al. 2009
*Gibberella intermedia* CA3-1	na	3-cyanopyridine	Wu et al. 2013
*Aspergillus niger* K10	*100*	Benzonitrile	Kaplan et al. 2006a
*Penicillium multicolor* CCF 2244	9.3	Benzonitrile	;Martínková et al. 2009
*Fusarium proliferatum* ZJB09150	na	3-cyanopyridine	Jin et al. 2012
*Fusarium proliferatum* AUF-2	2000	Benzonitrile	Communicated
*Fusarium proliferatum* AUF-2	>4000	Benzonitrile	**Present work**

Keeping this in view, we screened out an efficient nitrilase producing fungal strain from *Fusarium proliferatum* (communicated). This strain has shown promising nitrilase activity with broad substrate specificity. This strain is a pure nitrilase producer i.e. produces only acids from corresponding nitriles with no amide formation as side product. Thus, this is more specific as compared to the previously reported nitrilase from *Fusarium proliferatum* (Jin et al. 2012)*.* Moreover, our strain *Fusarium proliferatum* AUF-2 represented second highest nitrilase activity (>2000U/L culture) after *Fusarium solani* producing nitrilase activity of >3000U/L culture (Vejvoda et al. 2008). We, therefore, decided to optimize growth conditions and production medium to maximize nitrilase production.

Optimization of enzyme production through manipulation of suitable medim components by conventional methods is quite tedious, time consuming and costly process (Mason et al. 2003). The concept of design of experiments (DOE) makes the optimization easy, helps in building the models, evaluating the effect of several factors, achieving the optimum conditions for desirable responses and also reduced the number of experiments to large extent (Montgomery 2005). Analysis of variance (ANOVA) provides the statistical results and diagnostic checking tests which enables researchers to evaluate adequacy of the models (Ghafari et al. 2009). RSM is a collection of mathematical and statistical method which helps in studying the effects of various parameters at a single time, is faster to implement, time saving and cost effective (Montgomery 2005; Layh et al. 1977). Various studies have recently been reported on optimization of nitrilase production in bacterial strains using RSM (Dubey et al. 2011; Shen et al. 2009). However, to the best of our knowledge, optimization of nitrilase production from fungal strains using RSM has not been reported.

In the present study, we have studied nitrilase production in this strain by medium optimization through RSM. Three independent variables chosen for the study were the carbon source (glucose), nitrogen source (sodium nitrate) & inducer (ϵ-caprolactam) and the response value for nitrilase activity was chosen as the dependent variable.

## Materials and methods

### Strain and growth conditions

Nitrilase producing *Fusarium proliferatum*, strain AUF-2 (NCBI Accession no. JX840861) was isolated from the soil sample of Shivalik region, India and was maintained on a modified Czapek-Dox agar medium. Medium composition in g/l was: sucrose 30, NaNO_3_ 3.0, K_2_HPO_4_ 1.0, MgSO_4_·7H_2_O 0.5, KCl 0.5, FeSO_4_·7H_2_O 0.01, CoCl_2_·6H_2_O 0.001, ZnSO_4_·7H_2_O 0.0067; pH 7.0 with 20 g/l agar. Constituents of the seed (without inducer) and production medium (with nitrile inducer; ϵ-caprolactam) were the same without agar. The seed medium was inoculated from a freshly cultured slant and incubated under shaking conditions in an orbital shaker at 200 rpm for 48 h at 28°C. 5% of the seed was transferred to production medium and was grown for 72 h under same conditions. Biomass was harvested by centrifugation at 15,000 × *g* for 15 min at 4°C. The cell pellet was washed twice with 0.1 M phosphate buffer (pH 8.0) for further use.

### Nitrilase enzyme assay

Nitrilase activity of the fungal enzyme was assayed by measuring the production of ammonia during the hydrolysis of benzonitrile to benzoic acid. 10 mg of wet cell biomass was incubated in the presence of benzonitrile (30 mM) followed by estimation of amount of ammonia released by using the method of Fawcett & Scott (1960). One unit (U) of nitrilase is defined as the micromoles of ammonia released per min under standard assay conditions i.e. pH 8.0; 37°C by cell biomass. Enzyme activity of whole cells was calculated by the following formula:

### Optimization for nitrilase activity by One-variable-at-a-time approach

The selection of medium components including carbon sources, nitrogen sources and inducers were investigated through the traditional ‘one-variable- at-a-time’ approach. Effect of various carbon sources, nitrogen sources and inducers were tested to evaluate their effect on nitrilase production, while other ingredients were kept unchanged (communicated). The concentrations of these factors for zero coded levels of variables in the subsequent optimization were confirmed by single parameter optimization studies.

### Experimental design and optimization by RSM

Based on single parameter optimization studies carbon source (glucose), nitrogen source (sodium nitrate) and inducer (ϵ-caprolactam) were found to be the suitable medium components for optimum nitrilase production. RSM and CCD were used to optimize the response of these three variables by statistical software “Design Expert-8.0.7.1”. The program generated twenty experiments (eight factorial, six axial and six central). The experiments were carried out in random order with six replicates at the central point to calculate the pure error of the model. The experimental results of CCD were fitted with a second-order polynomial equation as shown below:

where, Y is the nitrilase activity (U/g biomass); A is the coded value of glucose concentration; B is the coded value of sodium nitrate concentration and C is the coded value of ϵ-caprolactam concentration. a_0_ is a constant, a_1_, a_2_ and a_3_ were linear coefficients, a_11_, a_22_ and a_33_ were squared coefficients and a_12_, a_13_ & a_23_ were interaction coefficients. Response surface graphs were plotted to determine maximum nitrilase activity.

## Results

### Optimization of carbon source, nitrogen source and inducer concentration by one variable-at-a-time approach

Our previous studies (communicated) reveal that three variables namely carbon source, nitrogen source and inducer are the most significant constituents responsible for nitrilase production. It was also found that our strain *Fusarium proliferatum* AUF-2 produces maximum nitrilase (26U/g biomass) with glucose as carbon source, sodium nitrate as nitrogen source and ϵ-caprolactam as inducer. We therefore, optimized the concentrations of these three independent variables using One-variable-at-a-time Approach. Optimization results revealed that 50 g/l glucose, 2.5 g/l sodium nitrate and 3.5 g/l ϵ-caprolactam would result in maximum nitrilase enzyme production (56.8 U/g). These values were therefore selected as the zero coded values of glucose, sodium nitrate and ϵ-caprolactam respectively (Table 2) for further optimization using RSM.

**Table 2:** **Independent variables selected for experimental design**

Factors	Symbols	Code levels		
		−1	0	+1
Glucose (g/l)	A	40	50	60
Sodium nitrate (g/l)	B	2.0	2.5	3.0
ϵ-Caprolactam (g/l)	C	2.5	3.5	4.5

### Fit summary for model fitting and ANOVA

The values of three variables i.e. glucose, sodium nitrate and ϵ-caprolactam with the actual and predicted nitrilase activity (U/g) in 20 random experimental runs are shown in Table 3. The second order polynomial model was used to correlate the independent variables with nitrilase activity. The best candidate to fit the data was the quadratic model after fit summary comparison (*p*<0.0001).

**Table 3:** **Central composite design matrix for the experimental and predicted results in the production of nitrilase**

Run	Glucose (g/l)	Sodium nitrate (g/l)	ϵ-caprolactam (g/l)	Predicted activity(U/g)	Actual activity(U/g)
1	50.00	1.66	3.50	48.65	48 ±2.06
2	50.00	2.50	1.82	32.03	30 ±2.24
3	66.82	2.50	3.50	54.20	50 ±1.35
4	50.00	2.50	3.50	58.14	59 ±1.24
5	33.18	2.50	3.50	51.57	49 ±2.35
6	40.00	3.00	4.50	42.88	44 ±1.32
7	40.00	3.00	2.50	44.77	45 ±1.23
8	50.00	3.34	3.50	46.12	46 ±1.42
9	60.00	3.00	2.50	44.33	45 ±1.34
10	50.00	2.50	3.50	58.14	56 ±2.33
11	40.00	2.00	2.50	38.27	41 ±2.31
12	50.00	2.50	5.18	34.74	30 ±3.24
13	60.00	2.00	2.50	44.33	48 ±2.52
14	50.00	2.50	3.50	58.14	55 ±3.26
15	40.00	2.00	4.50	45.88	50 ±3.94
16	50.00	2.50	3.50	58.14	62 ±2.63
17	60.00	2.00	4.50	49.44	54 ±2.45
18	50.00	2.50	3.50	58.14	60 ±1.38
19	60.00	3.00	4.50	39.94	42 ±1.33
20	50.00	2.50	3.50	58.14	58 ±1.23

As shown in Table 4, the quadratic model was found to be the best fit model using sum of square analysis which compares the ratio of mean square regression to the mean square residuals. A low value (7.35%) of the coefficient of variance (CV) indicates a very high degree of precision and good reliability of experimental values. Both the R^2^ value (0.91) and the adjusted R^2^ value (0.83) were high in this model indicating the fit of model. Adequate Precision for our model has a signal-to-noise ratio of 10.584 which indicates an adequate signal.

**Table 4:** **Model fitting values**

Model terms	Values
CV (%)^a^	7.35
R^2 b^	0.91
Adjusted R^2 c^	0.83
Adequate precision ^d^	10.58
Standard deviation	3.57

^a^ CV (%): coefficient of variance, is the standard deviation which is expressed as a percentage of the mean.

^b^ R^2^: is the measure of variation around the mean explained by the model.

^c^ Adjusted R^2^: is the measure of variation around the mean explained by the model, adjusted for the number of terms in the model. The adjusted R^2^ decreases as the number of terms in the model increases provided those additional terms do not add value to the model.

^d^ Adequate precision: compares the range of predicted value at the design points to the average prediction error.

Table 5 shows the ANOVA of the best fitted quadratic model. It has three factors having nine significant variances (A, B, C, AB, AC, BC, A^2^, B^2^ and C^2^). Each variance has been evaluated by ANOVA. The Model F-value of 11.79 implies that the model is significant. There is only a 0.03% chance that a “Model F-Value” could occur due to noise. The ANOVA table shows only the significant variances i.e. B (nitrogen source, NaNO_3_), C (inducer, ϵ-caprolactam), B^2^ (quadratic terms of NaNO_3_, p value of 0.0103) and C^2^(quadratic terms of ϵ-caprolactam, p value of <0.0001).

**Table 5:** **Analysis of variance (ANOVA) for response surface quadratic model**

Source	Sum of squares	df	Mean square	F-value	P-value prob > F
Model	1355.14	9	150.57	11.79	0.0003
Lack of fit	94.33	5	18.87	2.83	0.1391
B- Sodium nitrate	30.36	1	30.36	2.38	0.1540
C- Caprolactam	8.86	1	8.86	0.69	0.4242
B^2^	126.67	1	126.67	9.92	0.0103
C^2^	1160.86	1	1160.86	90.93	<0.0001
Pure error	33.33	5	6.67		
Cor total	1482.80	19			

**Figure 1:**
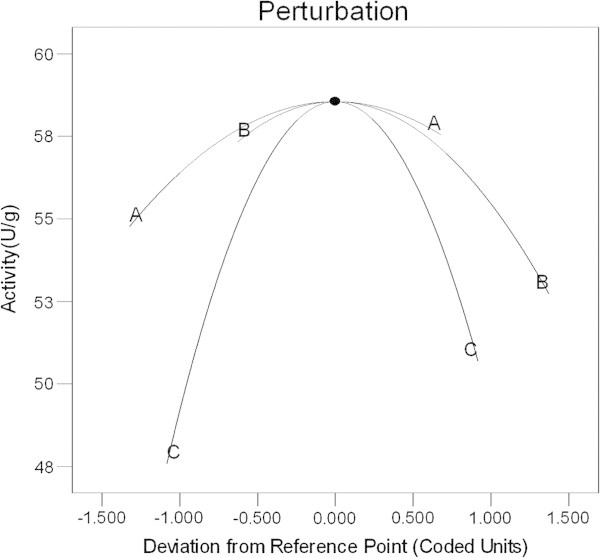
**Perturbation Plot, A (glucose), B (sodium nitrate) and C (ϵ-caprolactam).**

The perturbation plot (Figure 1) compared the effect of all the factors at a particular point in the design space. A perturbation plot at the centre point (glucose 53.22 g/l, sodium nitrate 2.31 g/l, caprolactam 3.58 g/l) was obtained to show the relative effect of the three chosen variables as ‘one factor at a time’ on nitrilase production. The perturbation plot indicated that inducer (C) has the most influential effect (steepest slope) on nitrilase production followed by nitrogen source (B), whereas, carbon source (A) has the least effect on nitrilase production.

### Final equation and model graphs

The values of regression coefficients were calculated and the experimental results of CCD were fitted with second order polynomial equation. The predicted values of nitrilase production (Y) for 20 independent experiments were calculated by the following equation with actual values of glucose (A), sodium nitrate(B), and ϵ-caprolactam(C).

**Figure 2:**
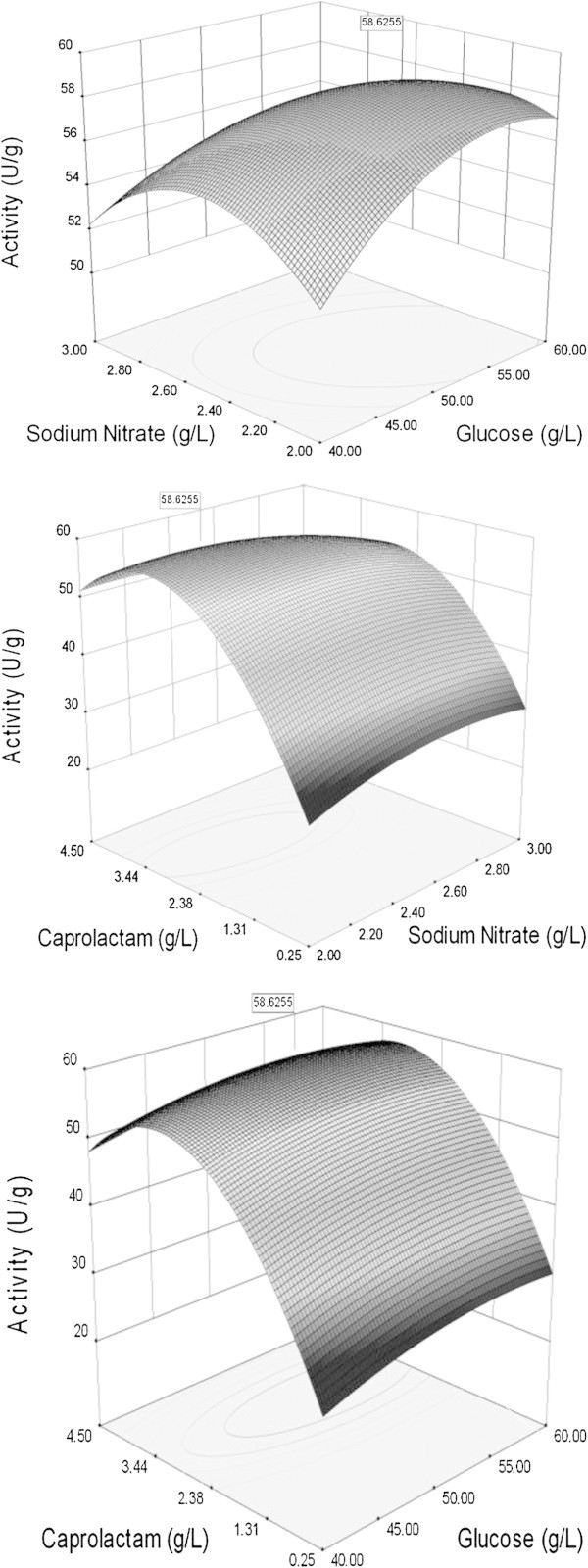
**Three dimensional contour plots showing the effect of different variables on the nitrilase production by*Fusarium proliferatum*.
**

Based on the above equation, predicted value of nitrilase activity at any variable concentration can be calculated. Figure 2 represents three dimensional surface plots showing the combined effect of two independent variables for nitrilase production, while the third variable was kept at zero coded value.

### Optimization and validation

**Figure 3:**
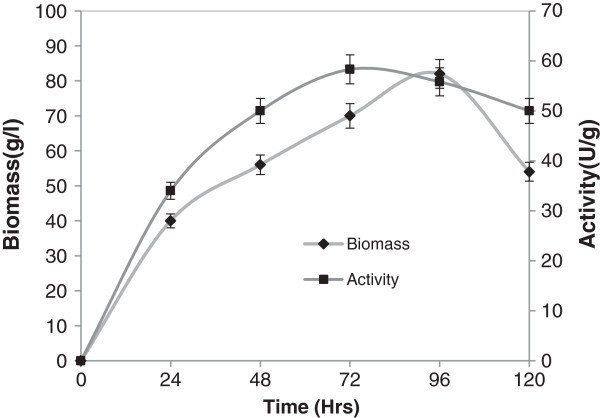
**The growth curve of*Fusarium proliferatum*and nitrilase activity (U/g biomass) after RSM optimization.**

Based on the model, optimum medium composition was obtained at 53.22 g/l glucose, 2.31 g/l sodium nitrate and 3.58 g/l ϵ-caprolactam concentration resulting in predicted nitrilase activity of 59.0 U/g biomass. Validation of the predicted results was done under optimized conditions in three independent experiments. Figure 3 shows the average of results obtained in these experiments. It can be seen that the experimental nitrilase activity of 58.3 U/g was obtained which correlated to the predicted activity (59.0 U/g) confirming the rationality of the model. This is 2.24 fold higher than that obtained before optimization (26.0 U/g). Thus, overall 2.24 fold increase in nitrilase activity was observed after optimization using RSM. Comparison of fungal strains reported in literature for nitrilase production has been shown in Table 1. Present work resulted in an overall production of nitrilase (>4000 U/l culture) as compared to the yield of (>2000 U/l culture) obtained before optimization.

## Discussion

Enzymatic conversion of nitrile to corresponding acids has been one of the most interesting biotransformations during the past few years, although nitrilase catalysed reactions are still unexplored. Therefore, nitrilase production through medium optimization by fast and effective experimental design is essential. It has been reported that growth and enzyme production can be enhanced to several folds by a statistics based experimental design. Medium selection and its components during fermentation at shake flask level has shown remarkable enhancement in the activity of various enzymes (Montgomery 2005; Myers et al. 2002; Layh et al. 1977). Statistical experimental design viz. multivariate designs, allows multiple control variables, is faster to implement and cost effective as compared to traditional univariate approach. As far as nitrilases are concerned, enhancement of nitrilase production using RSM has been studied with only bacterial source (Dubey et al. 2011; Shen et al. 2009) and fungi have not been studied in this context.

It has been shown that there are only three main factors namely carbon source, nitrogen source and inducer which can affect nitrilase activity (Gong et al. 2012). In order to enhance the enzyme production by design of experiments, we chose three important medium components viz. glucose (carbon source), sodium nitrate (nitrogen source) and ϵ-caprolactam (inducer). Thus, during the present study, we have optimised these three medium components in order to achieve maximum nitrilase production from *Fusarium proliferatum* AUF-2*.* In order to understand the effect of these three independent variables, Perturbation plot was generated from one factor at a time experiments (Figure 1). The x-axis shows a deviation of the factor values from the reference point as actual value and y-axis shows nitrilase activity (U/g). Steepest curvature in case of variable C (inducer) depicts that the response (nitrilase activity) is sensitive to inducer concentration. The trend of the perturbation plot was in agreement with the previous observations by Dubey et al. (2011) and Shen et. al. (2009). The results indicate that inducer is the most significant variable, whereas carbon source is the least significant variable for nitrilase production. These results are in agreement with the previous work showing that nitrogen source and inducer have direct relation with nitrilase activity, whereas carbon source is responsible for biomass production (Shen et al. 2009). The results also support the literature that most of the nitrilases are inducible in nature (Martínková et al. 2009).

As shown in Table 4, the quadratic model was found to be the best fit model using sum of square analysis which compares the ratio of mean square regression to the mean square residuals. P<0.05 indicates that model terms are significant. In this case B,C, B^2^, C^2^ are significant model terms indicating that sodium nitrate and ϵ-caprolactam have significant effect on nitrilase production. Model F value of 11.79 for nitrilase production implies significant model and “Lack of Fit” F value of 2.83 implies insignificant Lack of Fit. The coefficient of variance (CV%) value of 7.35 indicates a very high degree of precision and good reliability of experimental data. The coefficient of determination (R^2^) should always be closer to 1.00. Higher and close to 1.0 value of R^2^ (0.91) in this model indicates that a high percentage of the variability in the response could be explained by the model. This also implies satisfactory adjustment of quadratic model to the experimental data. Adjusted R^2^ value (0.83) indicated a good agreement between the experimental and predicted values for nitrilase production. Adjusted R^2^ which is smaller than R^2^ indicates that too many terms are present in the model ratio (Haaland 1989). Statistical analysis also determines the experimental factors that generate signals, large enough as compared to the noise. Adequate Precision measures the signal to noise ratio (Haaland 1989) and a ratio greater than 4 is desirable. Our model has a ratio of 10.584 which indicates an adequate signal.

Based on the results obtained with “one factor at a time approach”, RSM was used to build the model. The model helps to study the interactions between the variables i.e. medium components and the optimum concentration of each component. The three dimensional response surface plots were constructed by plotting the nitrilase activity on the Z axis against two independent variables with third variable being at fixed level. This allowed to understand the significance of model during interaction of the medium components and optimum concentration of each medium component for nitrilase production. Analysis of variance (ANOVA) showed that medium component B (sodium nitrate), medium component C (ϵ-caprolactam), B^2^, C^2^ are significant model terms. Figure 2 shows the three dimensional response surface plots for interaction between glucose-sodium nitrate, sodium nitrate- ϵ-caprolactam and glucose-ϵ-caprolactam. Analysis of response surface plots indicated that sodium nitrate and ϵ-caprolactam slight variation in concentrations of these components from zero code value led to an increase in nitrilase activity. Based on the model, optimum medium composition was observed at 53.22 g/l glucose, 2.31 g/l sodium nitrate and 3.58 g/l ϵ-caprolactam with a predicted nitrilase activity of 59.0U/g biomass.

Verification of predicted results was accomplished by three independent experiments carried out with optimised medium components viz. 53.22 g/l glucose, 2.31 g/l sodium nitrate and 3.58 g/l ϵ-caprolactam. The results obtained as an average (58.3 U/g nitrilase activity) were in close agreement with statistically predicted value (59.0 U/g), confirming the authenticity of the model. After optimization, a 2.24 fold increase in nitrilase production was observed as compared to the initial activity of 26.0 U/g. These results indicate that the optimization of medium component through RSM favoured enhanced nitrilase production providing overall yield of >4000 U/l culture.

In conclusion, second order quadratic model was found to fit the experimental data obtained from RSM using three key medium components glucose (carbon source), sodium nitrate (nitrogen source) and ϵ-caprolactam (inducer). It has been found that the inducer i.e. ϵ-caprolactam is the most significant variable for nitrilase production. Validation experiments of optimal conditions showed 2.24 fold increase in nitrilase production resulting in 58.3 U/g nitrilase activity in *Fusarium proliferatum* strain.

## Authors’ contribution

FY and AC planned the experiments. FY carried out the experimental work. UJ isolated and maintained the culture and helped in carrying out experiments. AR helped in experimentation and data analysis. RP provided the facilities and resources for carrying out the experiments. AC supervised the work and prepared the manuscript. All authors read and approved the final manuscript.

## Abbreviations

ANOVA: Analysis of variance
CCD: Central composite design
RSM: Response surface methodology
DOE: Design of experiments
CV (%): Coefficient of variance
R2: Coefficient of determination.

## References

[CR1] Babu V, Shilpi CB (2010). Nitrile-metabolizing potential of *Amycolatopsis* sp. IITR215. Process Biochem.

[CR2] Banerjee A, Sharma R, Banerjee UC (2002). The nitrile-degrading enzymes: current status and future prospects. Appl Microbiol Biotechnol.

[CR3] Dubey S, Singh A, Banerjee UC (2011). Response surface methodology of nitrilase production by recombinant *Escherichia coli*. Braz J Microbiol.

[CR4] Fawcett JK, Scott JE (1960). A rapid and precise method for the determination of urea. J Clin Path.

[CR5] Ghafari S, Aziz HA, Isa MH, Zinatizadeh AA (2009). Application of response surface methodology (RSM) to optimize coagulation–flocculation treatment of leachate using poly-aluminum chloride (PAC) and alum. J Hazard Mater.

[CR6] Gong JS, Lu ZM, Li H, Shi JS, Zhou ZM, Xu ZH (2012). Nitrilases in nitrile biocatalysis: recent progress and forthcoming research. Microb Cell Fact.

[CR7] Gupta V, Gaind S, Verma PK, Sood N, Srivastava AK (2010). Purification and characterization of intracellular nitrilases from *Rhodococcus* sp.- potential role of periplasmic nitrilase. Afr J Microbiol Res.

[CR8] Haaland PD, Haaland PD (1989). Statistical problem solving. Experimental Design in Biotechnology.

[CR9] Jin LQ, Liu ZQ, Xu JM, Zheng YG (2012). Biosynthesis of nicotinic acid from 3-cyanopyridine by a newly isolated Fusarium proliferatum ZJB-09150. World J Microbiol Biotechnol.

[CR10] Kaplan O, Vejvoda V, Plíhal O, Pompach P, Kavan D, Bojarová P, Bezouška K, Macková M, Cantarella M, Jirků V, Křen V, Ludmila Martínková L (2006a). Purification and characterization of a nitrilase from Aspergillus niger K10. Appl Microbiol Biotechnol.

[CR11] Kaplan O, Vejvoda V, Charvátová-Pinvejcová A, Martínková L (2006b). Hyperinduction of nitrilases in Wlamentous fungi. J Ind Microbiol Biotechnol.

[CR12] Kobayashi M, Shimizu S (2000). Nitrile hydrolases. Curr Opin Chem Biol.

[CR13] Layh N, Hirrlinger B, Stolz A, Knackmuss HJ (1977). Enrichment strategies for nitrile- hydrolysing bacteria. Appl Microbiol Biotechnol.

[CR14] Marron AO, Akam M, Walker G (2012). Nitrile hydratase genes are present in multiple eukaryotic multiple supergroups. PLoSOne.

[CR15] Martínková L, Vejvoda V, Kaplan O, Kubáč D, Malandra A, Cantarella M, Bezouška K, Křen V (2009). Fungal nitrilases as biocatalysts: recent developments. Biotechnol Adv.

[CR16] Mason RL, Gunst RF, Hess JL (2003). Statistical Design and Analysis of Experiments, Eighth Applications to Engineering and Science.

[CR17] Montgomery DC (2005). Design and analysis of experiments.

[CR18] Myers RH, Montgomery DC, Anderson Cook CM (2002). Response surface methodology: process and product optimization using designed experiments.

[CR19] O’Reilly C, Turner PD (2003). The nitrilase family of CN hydrolysing enzymes – a comparative study. J Appl Microbiol.

[CR20] Petříčková A, Veselá AB, Kaplan O, Kubáč D, Uhnáková B, Malandra A, Felsberg J, Rinágelová A, Weyrauch P, Křen V, Bezouška K, Martínková L (2012). Purification and characterization of heterologously expressed nitrilases from filamentous fungi. Appl Microbiol Biotechnol.

[CR21] Schreiner U, Hecher B, Obrowsky S, Waich K, Klempier N, Steinkellner G, Gruber K, Rozzell JD, Glieder A, Winkler M (2010). Directed evolution of *Alcaligenes faecalis* nitrilase. Enzyme Microb Technol.

[CR22] Sharma NN, Sharma M, Bhalla TC (2011). An improved nitrilase-mediated bioprocess for synthesis of nicotinic acid from 3-cyanopyridine with hyperinduced *Nocardia globerula* NHB-2. J Ind Microbiol Biotechnol.

[CR23] Shen M, Liu ZQ, Zheng YG, Shen YC (2009). Enhancing Endo-nitrilase production by a newly isolated *Arthrobacter nitroguajacolicus* ZJUTB06-99 through optimization of culture medium. Biotechnol Bioproc Eng.

[CR24] Thuku RN, Brady D, Benedik MJ, Sewell BT (2009). Microbial nitrilases: versatile, spiral forming, industrial enzymes. J Appl Microbiol.

[CR25] Vejvoda V, Kaplan O, Bezouška K, Pompach P, Sulc M, Cantarella M, Benada O, Uhnakova B, Rinagelova A, Lutz-wahl S, Fischer L, Kren V, Martinkova L (2008). Purification and characterization of a nitrilase from *Fusarium Solani* O1. J Mol catal B: Enz.

[CR26] Vejvoda V, Kubaˇc D, Davidova A, Kaplan O, Sulc M, Sveda O, Chaloupkova R, Martinkova L (2010). Purification and characterization of nitrilase from Fusarium solani IMI196840. Process Biochem.

[CR27] Wu Y, Gong J, Lu Z, Li H, Zhu XY, Li H, Shi JS, Xu ZH (2013). Isolation and characterization of *Gibberella intermedia* CA3-1, a novel and versatile nitrilase-producing fungus. J Basic Microbiol.

[CR28] Xue YP, Xu SZ, Liu ZQ, Zheng YG, Shen YC (2011). Enantioselective biocatalytic hydrolysis of (R, S)-mandelonitrile for production of (R)-(2)-mandelic acid by a newly isolated mutant strain. J Ind Microbiol Biotechnol.

[CR29] Zhu D, Mukherjee C, Yang Y, Rios BE, Gallagher DT, Smith NN, Biehl ER, Hua L (2008). A new nitrilase *from Bradyrhizobium japonicum* USDA 110: gene cloning, biochemical characterization and substrate specificity. J Biotechnol.

